# Understanding the sources, function, and irreplaceable role of cobamides in organohalide-respiring bacteria

**DOI:** 10.3389/fmicb.2024.1435674

**Published:** 2024-07-30

**Authors:** Yongfeng Lu, Fancheng Lu, Jian Zhang, Qianwei Tang, Dan Yang, Yaqing Liu

**Affiliations:** ^1^College of Light Industry and Food Engineering, Guangxi University, Nanning, China; ^2^College & Hospital of Stomatology, Guangxi Medical University, Nanning, China; ^3^Guangxi Yuhuacheng Environmental Protection Technology Co., Nanning, China

**Keywords:** OHRB, reductive dehalogenation, cobamides, salvage pathway, co-culture

## Abstract

Halogenated organic compounds are persistent pollutants that pose a serious threat to human health and the safety of ecosystems. Cobamides are essential cofactors for reductive dehalogenases (RDase) in organohalide-respiring bacteria (OHRB), which catalyze the dehalogenation process. This review systematically summarizes the impact of cobamides on organohalide respiration. The catalytic processes of cobamide in dehalogenation processes are also discussed. Additionally, we examine OHRB, which cannot synthesize cobamide and must obtain it from the environment through a salvage pathway; the co-culture with cobamide producer is more beneficial and possible. This review aims to help readers better understand the importance and function of cobamides in reductive dehalogenation. The presented information can aid in the development of bioremediation strategies.

## Introduction

Organohalides exist on earth with volcanic activity, forest fire, organic oxidation, and animal or plant activity (Chandra and Kumar, 2015). With the development of the chemical industry, many artificially complex organohalides have been synthesized and produced for commercial use. The long-term use and improper disposal of organohalides (e.g., pesticides, chemical reagents, and industrial activities) cause widespread pollution (Gribble, 1998; Jin and Chen, 2017; Kallenborn et al., 2021; Xu et al., 2021). Typical organohalides include perchloroethylene (PCE), hexachlorobenzene (HCB), trichloroethane (TCA), polychlorinated biphenyls (PCBs), pentachlorophenol (PCP), hexachlorocyclohexanes (HCHs), dichlorodiphenyltrichloroethanes (DDTs), polybrominated diphenyl ethers (PBDEs), poly-fluoroalkyl substances (PFASs), and so on (Huang et al., 2014; Lu et al., 2019; He et al., 2021; Abbasian Chaleshtari and Foudazi, 2022; Cheng et al., 2023). Besides the persistence and stability in the environment, its carcinogenicity, teratogenicity, and mutagenicity may irreversibly damage the balance of the ecosystem and pose a carcinogenic risk to humans (Yankovych et al., 2023). Moreover, most organohalides are lipophilic, which allows them to stay in fat-rich organs, and are difficult to metabolically eliminate. Their long-term accumulation may damage the nervous and immune systems (Bennett et al., 2021).

Among the various remediation strategies for organohalide contamination, traditional physical and chemical methods, such as incineration, adsorption, advanced oxidation, and electrochemical techniques, can effectively remove or degrade organohalide contamination (Xu et al., 2021; Femina Carolin et al., 2023). However, these methods pose challenges due to the by-products, high costs, and energy consumption. In contrast, biotransformation has emerged as an economically and environmentally friendly method for eliminating organohalides from the environment rather than transferring the organohalide contamination to another location (Zhu et al., 2022). Microorganisms play a crucial role in conducting bioremediation processes, with many having the ability to transform organohalide pollutants, e.g., *Dehaloococcoides, Dehalobacter, Desulfitobacterium, Geobacter*, and *Sulfurospirillum* (Villemur et al., 2006; Maillard and Holliger, 2016; Zinder, 2016; Reguera and Kashefi, 2019; Jin et al., 2023), which possess a unique reductive dehalogenase (RDase); they are known as organohalide-respiring bacteria (OHRB). During the transformation process, OHRB use organohalides as an electron acceptor and drives free electron transport to RDase, which leads to the bond cleavage of C-Cl. RDase functions as the terminal enzyme in the organohalide dehalogenation process and is crucial for halogen removal (Fincker and Spormann, 2017; Wang et al., 2018).

Previous studies confirmed that cobamide is integral to RDase. It enables RDase to react with organohalides and is important in OHRB metabolism (Yan et al., 2018). Cobamide, whose basic structure is a corrin ring, combines with an upper and lower ligand, forming a larger family of cobamide, such as cobalamin (Deery et al., 2022). Cobalamin is the most specific subunit of cobamide that is directly involved in removing halogen atoms (Kunze et al., 2017; Schubert et al., 2019). However, it is interesting to note that not all OHRB can synthesize cobamide to assemble functional RDase; therefore, the ability to synthesize cobamide can be considered a characteristic to distinguish corrinoid-auxotrophic OHRB (Maphosa et al., 2010). Similar to the distinction between facultative OHRB and obligate OHRB, most obligate OHRB are also corrinoid-auxotrophic OHRB; this characteristic is related to energy conservation. The more flexible metabolism of facultative OHRB allows them to grow on a variety of electron acceptors and easily establish co-metabolic relationships with other strains. In contrast, obligate OHRB are more restricted in obtaining energy, and organohalide respiration is their only energy-converse pathway (Zhang et al., 2021). Both obligate and facultative OHRB share the feature of using organohalide for their growth via RDase. Still, they have different suitability to cobamide and completely distinct ways of obtaining cobamide.

Facultative OHRB, such as *Geobacter lovleyi, Desulfovibrio, Sulfurospirillum multivorans*, and *Desulfitobacterium*, are described as containing a set of cobamide synthesis pathways, as well as genes for the transport/uptake (e.g., *btuBFCD*) of cobamide synthetic (e.g., *cbiZ, cbiB, cobU, cobS, cobT*) (Men et al., 2012; Reinhold et al., 2012; Schubert, 2017; Nakamura et al., 2018). However, most obligate OHRB are classified as corrinoid auxotrophs, including *Dehalobacter restrictus* spp., *Dehalococcoides mccartyi* spp., and *Dehalogenimonas* spp. In contrast, *Dehalobacter restrictus* Y51 has been identified to have a complete *de novo* corrinoid synthesis gene. However, the absence of the *cbiH* gene in *Dehalobacter restrictus* Y51 significantly affects corrinoid synthesis and is classified as a corrinoid-auxotrophic obligate OHRB. Similarly, *Dehalogenimonas lykanthroporepellens* BL-DC-9 has been found to lack the *cbi* gene, which prevents it from synthesizing corrinoid *de novo*. In addition, according to the Genbank data, species *Dehalococcoides mccartyi*, including strain 195, CBDB1, BAV1, VS, FL2, and GT, are characterized as typical corrinoid-auxotrophic OHRB without *de novo* corrinoid synthesis (Löffler et al., 2013). They cannot synthesize corrinoid, the basic structure of cobalamin for organohalide respiration (Yan et al., 2013). However, some studies have also suggested that corrinoid-auxotrophic OHRB have dehalogenation activity without producing cobalamin by themselves (Yan et al., 2016). It is plausible that the cobamides were synthesized by other symbiotic partners who provided these cobamides to OHRB for maintaining the dehalogenation process in a low-energy mode (Men et al., 2013; Yan et al., 2016).

The cobamide in biocatalysis has been studied for many years, and the effect of cobamides on OHRB has been reported for decades (Guo and Chen, 2018). However, the related information of cobamide on catalytic dehalogenation is still fragmented. In this review, the role of cobamide in OHRB is systematically summarized, including the impact of cobamides on organohalide respiration. In addition, cobamide as a catalyst in the dehalogenation process is discussed. We have also summarized the cobamide salvage pathway of OHRB and the synergy of cobamides in the microbial community. Additionally, we provide several suggestions for further investigations on cobamides for reductive dehalogenation and the applications of OHRB. This review also summarizes the role of cobamide in reductive dehalogenation and provides a reference for the study of reductive dehalogenation.

## Cobamide affects the metabolism of OHRB

**Figure 1:**
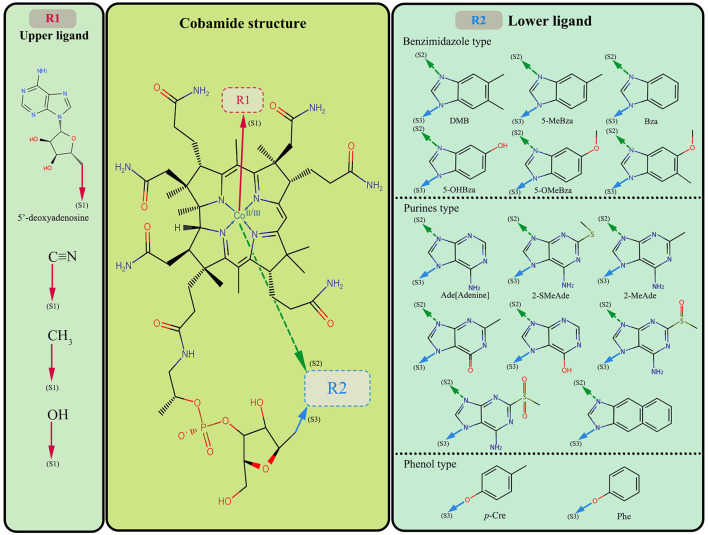
The middle part is the cobamide paradigm; R1 and R2 are the upper and lower ligands of the variable substructure, respectively. The left part lists the variable types of upper ligands and the right part accounts for the lower ligands of available cobamide. The red arrow is the connection position of the upper ligand and the cobamide structure, and the green and blue arrow represents the connection position of the lower ligand and the cobamide structure. DMB, 5,6-dimethylbenzimidazole; 5-MeBza, 5-methylbenzimidazole; Bza, benzimidazole; 5-OHBza, 5-hydroxybenzimidazole; 5-OMeBza, 5-methoxybenzimidazole; Ade, adenine; 2-SMeAde, 2-methylmercaptoadenine; 2-MeAde, 2-methyladenine; *p*-Cre, *p*-Cresol; Phe, Phenol.

The structure of cobamide consists of a corrin ring containing a central cobalt ion, a lower ligand, and an upper ligand; the ligand can be characteristic of various reported cobamide types in the natural environment. The typical lower ligand includes benzimidazole, purines, and phenol, and there are four common upper ligand types (Figure 1). Although the function of cobamide is mainly controlled by the lower ligand, previous research has shown that even cobamide forms with the same lower ligand type may not be functionally equivalent. Additionally, the upper ligand can also affect cobamide function (Zhai et al., 2012). It is widely accepted that cobalamin, with 5,6-dimethylbenzimidazole (DMB) as the lower ligand, is the most common cobamide type and is widely used (Yi et al., 2012).

The cyanogen group is the upper ligand of cobalamin (also called cyanocobalamin or vitamin B_12_), while the other upper ligand type includes hydroxyl, methyl, and 5′-deoxyadenosine, which can make up vitamin B_12_ analogs such as hydroxocobalamin, methylcobalamin, and 5′-deoxyadenosylcobalamin (Fang et al., 2017). The upper ligand also influence the cobamide function. Specifically, Zhai et al. (2012) suggested that cobalamin and hydroxocobalamin have no direct biological activity before biotransformation, and 5′-deoxyadenosylcobalamin is often applied as a coenzyme in microbial metabolism as it is the active coenzyme B_12_ form of cobalamin. The vitamin B_12_ analogues would be utilized after upper ligand biotransformation. It is more likely that metabolism converts the upper ligand to adenosine, enabling the cyanocobalamin into coenzyme B_12_, thus engaging in metabolic processes (Guo and Chen, 2018; Dulay et al., 2020). Here, we point out that the upper ligand may affect biological selectivity, but these effects are minor and not substantial enough to mask the influence of the lower ligand on biological selectivity. Therefore, we did not delve into the combined effects of cobamide upper ligand and lower ligand. Instead, we shifted our focus to discussing the lower ligand, and the following cobalamin generally refers to the cobalamin analogs.

The combination of the upper and lower ligands determines the variety of cobamide forms. It has been reported that 29 forms of cobamide exist in the environment (Brown, 2005), and at least 16 forms of lower ligand have been identified (Allen and Stabler, 2008; Yan et al., 2018). Each form of cobamide has its unique function in microbial metabolism. The lower ligand affects the transformation of cobamide into an active state, which causes different selections of cobamides in organisms (Yan et al., 2016). Based on the demand and synthesis of cobamide by community members, it can be divided into three types: those who do not use cobamide or produce it, those who use and produce cobamide alone, and those who need cobamide but are unable to produce it (Sokolovskaya et al., 2020). Shelton et al. (2019) estimate the proportion of these microorganism types in the environment: approximately 86% of bacteria require cobamides metabolism, but merely 37% produce cobamides *de novo*. The cobamide is assembled in many catalytic processes, including intramolecular rearrangements, methyl transfer, ribonucleotide reduction, and reductive dehalogenation (Dickman, 1977; Kunze et al., 2017; Farnberger et al., 2019). For example, the *Dehalococcoides mccartyi* is combined with the cobalamin catalysis. Cobalamin can be directly involved in the RDase assembly of *Dehalococcoides mccartyi*, whereas other cobamides require further remodeling through the cobamide salvage pathway (Yi et al., 2012). In addition, studies have reported that some OHRB have a preference for specific cobamide structures. For instance, *Sulfurospirillum multivorans* requires [Ade]cobamide (adenine as lower ligand) while the *Dehalococcoides mccartyi* strain prefers the cobalamin determined by the spatial structure of cobamide and RDase (Yi et al., 2012; Johannissen et al., 2017; Kunze et al., 2017).

The dehalogenation capability and the reaction rate are mostly affected by the lower ligand of cobamide, and specific lower ligand leads to high dehalogenation activity (Schubert et al., 2018). The activity of PceA from *Sulfurospirillum multivorans* was decreased, and the ability of organohalide respiration was also reduced after the DMB was replaced by adenine (Keller et al., 2014). Similarly, *Dehalococcoides mccartyi* did not show activity in dehalogenation during the cultivation without cobamide, while growth occurs when cobalamin or DMB structures is available (Yi et al., 2012; Men et al., 2015).

The cobamides in the natural environment are mainly produced by microbes (Men et al., 2015; Fang et al., 2017; Shelton et al., 2019), indicating that the forms of cobamides can be diverse in environments with complex microbial communities. As mentioned above, most cobamide cannot be assembled into RDase by OHRB directly, and suitable cobamide is limited. The endogenous production and exogenous utilization of cobamide can be the sources for OHRB. Endogenous cobamide biosynthesis is a complex process; except for some facultative OHRB, such as *Geobacter lovleyi*, that can synthesize cobamide to support dehalogenation, most OHRB cannot (Wagner et al., 2012; Deery et al., 2022).

For obligate OHRB, the lack of suitable cobamides restricts the application of exogenous cobamides. Even though they are the key factor for the RDase assembly process, not all available ones are used directly. The OHRB possess a set remodeling function that aims to transform these unsuitable cobamides into a directly usable form (Moore and Escalante-Semerena, 2016; Balabanova et al., 2021). This function involves the uptake of cobamides from the external environment and modification of the lower ligand to adapt to organohalide respiration, which is a complex process that may reduce dehalogenation efficiency (Men et al., 2014; Balabanova et al., 2021). The lower ligand mainly determines the effectiveness of cobamide function and influences the bioavailability of OHRB to different forms of cobamide. *Dehalococcoides mccartyi* strains have been discovered to modify at least seven cobamide lower ligands, including adenine, 2-SMeAde, 5-OHBza, Bza, 5-MeBza, *p*-Cresol, and corrinoid (Men et al., 2015). However, it should be noted that the remodeling function needs a lower ligand structure, such as DMB. The above remodeling function can only be achieved while DMB is present, even when the non-cobalamin forms are available. Generally, the addition of DMB enables guided cobalamin synthesis that supports RDase activity and OHRB growth (Yan et al., 2013, 2016).

The concentration and suitable structure of cobamides are both important for dehalogenation (Sokolovskaya Olga et al., 2019). According to previous reports, the concentration required by *Dehalococcoides* sp. for maintaining dehalogenation is 1 μg·L^−1^ (He et al., 2007). When the cobalamin concentration increased to 25 μg·L^−1^, the dehalogenation rate nearly doubled, the cell growth yield increased by 2.8–9.1 fold, and 50 μmol TCE was completely degraded to ethene in 1 month. The TCE degradation rates did not increase when the concentration of cobalamin was higher than 25 μg·L^−1^, which is also difficult to achieve in the environment. In the microcosm that contains cobalamin producers, approximately 10 μg·L^−1^ cobalamin was detected after cultivation, suggesting that the laboratory-calculated amount of minimum concentration (>1 μg·L^−1^) for OHRB growth is easy to achieve (Men et al., 2012). However, it should be noted that the concentrations of cobamide produced in the environment may differ from laboratory measurement; it may contain other available cobamides (such as 5-MeBza, 5-OHBza, etc.) that can also be used in dehalogenation. Thus, it is plausible that the growth of OHRB in the environment is not limited by cobamide but by the cobamide producers. Furthermore, to apply all the available cobamides for OHRB growth, a strategy is to use the remodeling function to transform these available cobamides into suitable ones. Yan et al. (2012) added 10 mM DMB into the culture to guide biosynthesis and generate cobalamin; compared to the control, the *Dehalococcoides mccartyi* strain BAV1, strain GT, and strain FL2 cell densities increased 31, 41, and 37 fold, respectively. In contrast, negligible *Dehalococcoides* growth was observed in the non-cobalamin cultures without DMB, which indicates the remodeling function of OHRB does not have sufficient DMB to catalyze the conversion of cobamide to cobalamin, leading to deficient RDase synthesis. Consequently, OHRB could not successfully obtain growth energy from organohalide respiration.

To conclude, in pure culture, the impact of cobamide on the dehalogenation process depends on two key factors: the lower ligand and the concentration of available cobamides. Alternatively, in the mixed culture, corrinoid-auxotrophic OHRB, especially, can perform more complete dehalogenation processes benefiting from the presence of other microorganisms that produce available cobamides and usable ligands.

## Cobamide as catalyst in dehalogenation

Since DeWeerd isolated and cultured the first OHRB in 1984, more than 70 OHRB strains have been identified in varied environments (Shelton and Tiedje, 1984; Atashgahi et al., 2016). The dechlorinating capability of different OHRB depends on the type of RDase that relies on the catalysis of cobamide (Ji et al., 2017). Different RDases obtain reducing power through the combination of cobamides, allowing RDase to undergo reductive dehalogenation. The high reducing power of cobamide also makes the OHRB vulnerable to oxygen, which requires strictly anaerobic conditions for their cultivation.

The potential application of OHRB for biotransformation is limited by their strict cultivation requirements and low energy yield (Wang et al., 2022). These factors also hinder the isolation and characterization of pure OHRB strains as well as the identification of the RDase. Many RDases were identified from *Dehalococcoides, Sulfurospirillum, Dehalobacter*, and so on, such as PceA, VcrA, and BvcA, were identified as degrading chlorinated ethenes (Neumann et al., 1996; Magnuson et al., 1998; Parthasarathy et al., 2015). DcaA, DcpA, and DcrA which catalyze the reductive dehalogenated of chlorinated hydrocarbons chlorinated hydrocarbons (Marzorati et al., 2007; Padilla-Crespo et al., 2013; Tang and Edwards, 2013); PcbA, PteA, and PbrA of reductive dehalogenated PBDEs or PCB (Ding et al., 2017; Zhao et al., 2021, 2022), and CbrA of reductive dehalogenated chlorobenzene (Monteagudo-Cascales et al., 2019).

**Figure 2:**
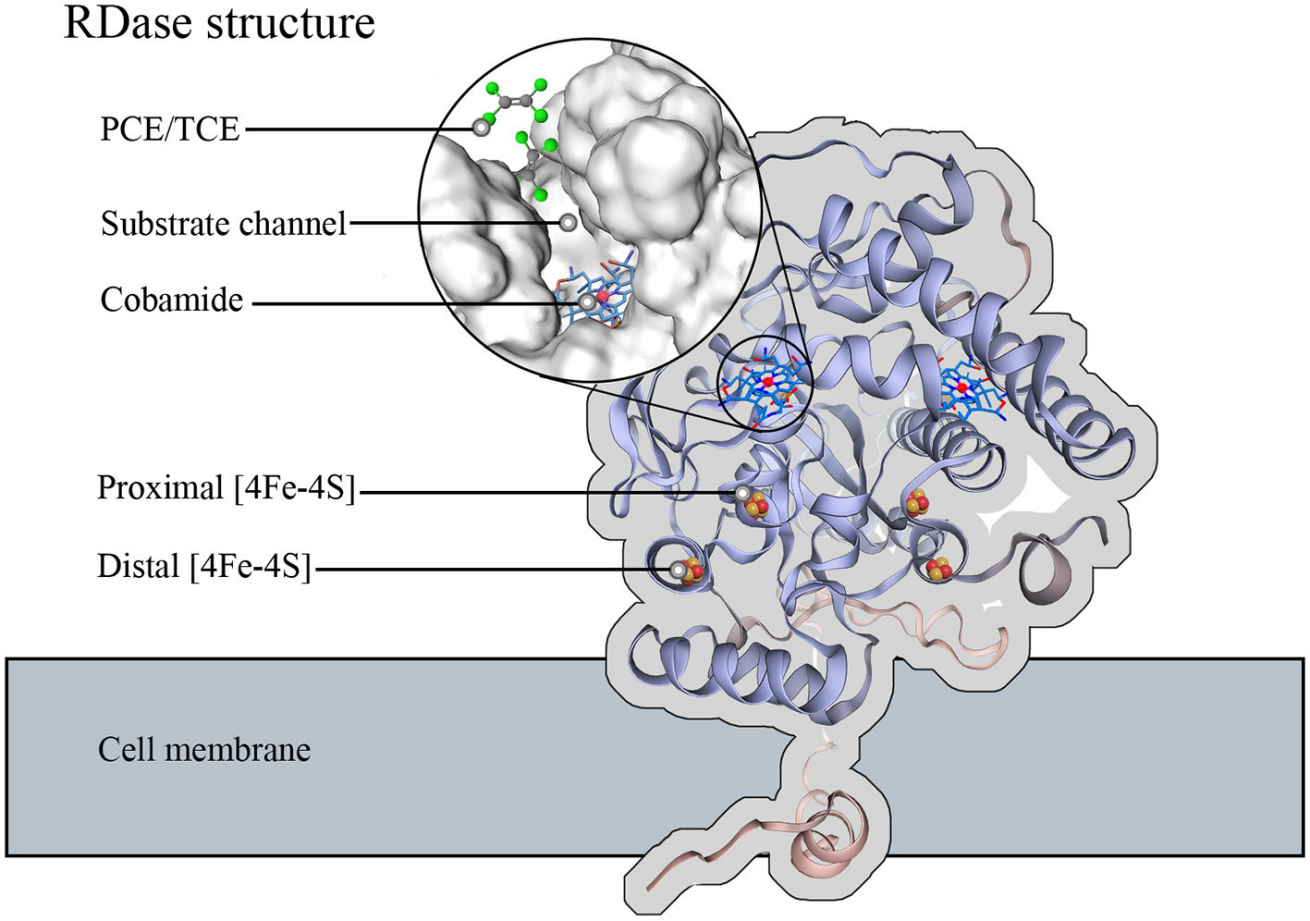
Function structure of most RDase. The RDase structure was used and simulated on SWISS-MODEL using the *Dehalococcoides mccartyi* dehalogenase gene provided by NCBI (GenBank: KSV18274.1) (Bienert et al., 2017; Waterhouse et al., 2018; Studer et al., 2020). The cobamide is embedded in the core of the RDase and accessible from substrate channels with two pair 4Fe-4S clusters that mediate electron transfer from the exterior to the cobamide.

Dehalogenation by different RDases involves a conservative electron transfer chain that is essential for organohalide respiration (Richardson, 2013). The hydrogenase catalyzes the oxidation of hydrogen or other electron donors to produce free electrons, which are then transferred through a series of electron-transport enzymes to the RDase (Kunze et al., 2017; Wang et al., 2018). For example, the free electrons through the quinone or CISM (Complex iron-sulfur molybdo) enzyme system to the RdhB (The membrane anchor protein), then the free electrons transmit to the RdhA (The membrane peripheral protein). In the internal structural electron transport of RDase (Figure 2), two pairs of 4Fe–4S clusters are identified in the RdhA. These were identified by electron paramagnetic resonance and UV-Vis spectroscopy (Parthasarathy et al., 2015; Nakamura et al., 2018). These two 4Fe-4S clusters are the final transit of the electron chain that transfers electrons to activate the cobamide. The corrin ring structure of cobamide provides six coordination bonds connecting the cobalt atoms, providing high reducibility for RDase. The substrate channel of RDase allows the organohalide to pass through and bind to the high-reducible cobamide (Jugder et al., 2015; Parthasarathy et al., 2015; Payne et al., 2015; Fincker and Spormann, 2017).

**Figure 3:**
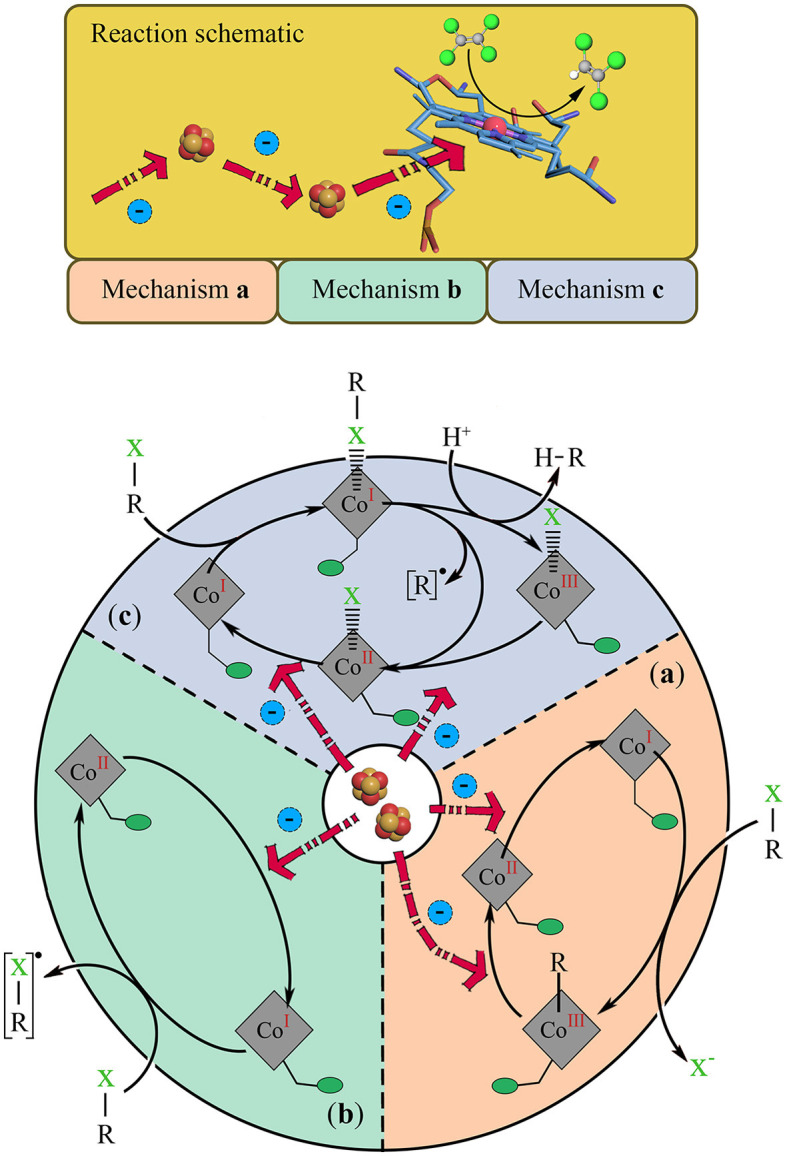
Cobamide structure in the dehalogenation reaction mechanism. The top part is the cobamide structure activated by the free electron transfer chain; and the bottom side are the possible mechanisms of cobalamin reacting with Organohalide (R-X), **(a)** the cobamide-organic adduct mechanism, **(b)** the free radical-catalytic mechanism, and **(c)** the cobamide-halide adduct mechanism. The cobamide state follows the reaction change and backtrack with the free electron activate, and finally form a closed cycle. The structure of 4Fe-4S cluster is shown by red and yellow balls, while PCE is shown as the green and gray balls.

As summarized in previous studies, the cobamide in RDase plays a crucial role in the reductive dehalogenation reaction (Table 1). The cobalamin receives free electrons transferred from the 4Fe-4S clusters to the cobalamin site, activating cobalamin and maintaining it in a highly reductive state, which activates the bond cleavage of the halogenated substrate in three ways: The first mechanism is the free radical-catalyzed dehalogenation mechanism (Figure 3b). The abstraction of an electron from the Co(I)balamin to substrate R-X forms the R-X radical state, and Co(II)balamin, the R-X radical, eliminates the halogen by further protonation/dehalogenation. Another free electron transfers from 4Fe-4S clusters to Co(II)balamin, leaving an unpaired electron on the cobalt center and reflash to Co(I)balamin. This exposes the cobalt's reducing ability, which allows it to react with various halogen radicals. The second mechanism is the cobamide-organic adduct mechanism (Figure 3a). The Co(I)balamin forms a bond with the carbon atom of the R-X, resulting in Co(III)balamin with the simultaneous elimination of halide ions and the subsequent cleavage of the C-Co bond under the electron transfer. At the same time, Co(III)balamin is reduced to Co(I)balamin by the electron transfer from the 4Fe-4S clusters. The third mechanism is the cobamide-halide adduct mechanism (Figure 3c). The Co(I)balamin directly interacts with a halogen atom of the substrate R-X, forming a Co(I)balamin-halide-carbon state. The halide-carbon bond then cleaves by protonation/radical action, forming a Co(III)/Co(II)balamin-halide state and reverting to the Co(I)balamin state through electron transfer.

**Table 1:** Previous research of cobalamin-mediated reductive dehalogenation.

**<Cobamide- mediated type**	**Reaction mechanism**	**Organohalide**	**RDase**	**Method**	**Reference**
Free radical-catalyzed-dehalogenation	NR	Trichloroethylene	PceA	Analog calculation	(Bommer et al., 2014)
Cobamide-halide adduct or Cobamide-organic adduct	Nucleophilic aromatic substitution or single electron transfer	Chlorinated and brominated aromatic	NR	NR	(Cooper et al., 2015)
Cobamide-halide-adduct	Heterolytic C-Br bond cleavage or homolytic C-Br bond cleavage	3,5-dibromo-4-hydroxybenzoic acid	NpRdhA	Analog calculation	(Payne et al., 2015)
Cobamide-halide-adduct	CoI-initiated concerted dehalogenation mechanism	2,6-dibromophenolate	NpRdhA	Analog calculation	(Liao et al., 2015)
Cobamide-halide adduct	[Co-X-R] adduct mechanism	2,6-dibromo-4-methylphenolate, 3,5-dibromo-4-hydroxybenzoic acid, tribromoethylene and trichloroethylene	NpRdhA, PceA	Analog calculation	(Johannissen et al., 2017)
NR	NR	Trichloroethylene	NR	Analog calculation	(Jin and Chen, 2017)
Cobamide-organic adduct	Addition–elimination or addition–protonation	Chlorinated ethenes	NR	Isotope fractionation	(Heckel et al., 2018)
Cobamide-halide adduct	Short-range or inner-sphere mechanisms	3,5-dibromo-4-hydroxybenzoic	NpRdhA	Analog calculation	(Halliwell et al., 2020)
Cobamide-organic adduct	Nucleophilic substitution-elimination mechanism	Chlorinated ethenes	VcrA, TceA, PteA	Isotope fractionation	(Franke et al., 2020)
Cobamide-organic adduct	Dihalo elimination and Nucleophilic Substitution	1,2-dibromoethane	*Dehalococcoides* spp.	Isotope fractionation	(Palau et al., 2023)

NR, not reported.

When the cobalt atom is transformed into the activated state of Co(I) through free electrons, it can provide a high reduction potential, which is crucial for reducing organohalides and is known as Co(I)balamin. Only this state is reactive as a low potential electron donor or nucleophilic reaction center, while the oxidation state of Co(II)balamin or Co(III)balamin is the oxidative state and possesses no reducibility. Furthermore, during the dehalogenation process, the standard potential of the cobalamin center depends on the RDase type. Wang et al. (2018) reviewed the redox potential of Co(II/I) in most of OHRB, and found that Co(II/I) transition *E*_m_ is often lower than −350 mV; the PceA of *Sulfurospirillum multivorans* has an estimated *E*° = −570 mV, similar to the CprA of *Dehalobacter restrictus* with *E*_*m*_ ≈ −480 mV; and the state of cobalamin is transformed from (I) to (II) and even to (III). This variation in the redox potential of cobalamin in different OHRB is significant as it influences the reactivity of cobalamin and, consequently, the dehalogenation process, and depends on the standard redox potentials of the organohalide substrates (*E*_*m*_: 260 to 570 mV) and the electron donors (e.g., $E_{0^{\prime }}$ of H_2_/H^+^ = −414 mV) (Wang et al., 2018; Yu et al., 2023). This requires the Fe-S clusters (*E*_*m*_ of 4Fe-4S = −440 mV) to transfer free electrons to revert the state back to Co(I)balamin (Fincker and Spormann, 2017). It should be noted that the actual electron transport process is more complex and requires various enzymes (Hase, dehydrogenase; quinone, intermediate electron shuttle; CISM; RdhB, etc.) to establish the electron transfer chain in the organohalide respiration. The bond cleavage mechanism and electron transfer chain in the organohalide respiration process still need further study.

## Salvage pathway of cobamide for OHRB

As discussed, specific cobamide form is an essential cofactor of RDase (e.g., cobalamin for *Dehalococcoides mccartyi* RDases, [Ade]cobamide for *Sulfurospirillum multivorans* PceA). It is crucial for the growth and energy conversion of these corrinoid-auxotrophic OHRB (Keller et al., 2018). The *Dehalococcoides* dehalogenation process seems to favor the cobalamin as the optimal cofactor, and these corrinoid-auxotrophic OHRB are more likely to take up the cobalamin than other cobamide when both are present. However, the corrinoid-auxotrophic OHRB are often deficient in the available cobalamin environment, or the main cobamide types are unsuitable for use. Compared to obligate OHRB, facultative OHRB are not restricted by organohalide respiration, which allows getting energy from other energy conversion metabolisms in the environment (Maphosa et al., 2010; Liu and Häggblom Max, 2018; Yang et al., 2020b; Liang et al., 2021; Zhang et al., 2022). Various metabolism pathways enable facultative OHRB to retain the function of corrinoid *de novo* synthesis and the organohalide respiration ability, such as *Geobacter lovleyi* (Nonaka et al., 2006; Wagner et al., 2012) and *Desulfitobacterium hafniense* Y51 (Reinhold et al., 2012; Schubert, 2017). In contrast, the low concentration of organohalides in the environment makes it hard to maintain obligate OHRB growth, and there is no extra energy to support the synthesis of corrinoids *de novo*. Consequently, some microorganisms, such as the species *Dehalococcoides mccartyi*, have been found to lack the gene for the synthesis of corrinoid *de novo* during evolution (Seshadri et al., 2005; Türkowsky et al., 2018). Shelton et al. (2019) suggests that the corrinoid *de novo* synthesis pathway consists of about 30 synthesis steps, which is more complex and redundant than the salvage pathway, consisting of several steps. Since these corrinoid-auxotrophic OHRB lack the genes for cobamide synthesis, they need alternative pathways to acquire cobamide for organohalide respiration (Rupakula et al., 2015; Moore and Escalante-Semerena, 2016).

The genome of species *Dehalococcoides mccartyi* has lost the genes for cobamide biosynthesis and replaced them with genes for cobamide modification and transport (Löffler et al., 2013; Yan et al., 2016; Men et al., 2017). In other words, the cobamide needed for its dehalogenation metabolism must be obtained from outside sources, and obtaining the necessary cobamide from other members of the dehalogenation community is often the most energy-efficient way. A previous study reported that the cobamide transport gene was detected in over 90% of OHRB (Zhang et al., 2009), and functional genes for cobamide uptake and salvage have been detected in typical corrinoid-auxotrophic *Dehalococcoides* species (Men et al., 2013). Further studies have shown that corrinoid-auxotrophic OHRB regulate genes involved in cobamide uptake and salvage when performing organohalide respiration (Men et al., 2014). Such as the BtuFCD protein responsible for the transport of cobalamin, the DMB phosphoribosyl transferase (CobT) and the adenosylcobinamidephosphate guanylyltransferase (CobU) to remodel the other cobamide to cobalamin (Escalante-Semerena, 2007; Balabanova et al., 2021; Ewald et al., 2022; Mathur et al., 2022). Additionally, all species of *Dehalococcoides mccartyi* possess genes such as *cbiP, cbiB, cobU, cobC, cobT*, and *cobS*, which are involved in cobalamin remodeling (Scott and Roessner, 2002; Wang et al., 2022). Furthermore, the polymerase chain reaction (PCR) amplification research showed that the defect of the cobamide synthesis gene will trigger the activation of the gene that regulates the transport and remedial pathway of cobamide (Moore and Escalante-Semerena, 2016). The transporter proteins are assembled in the cell and are used to identify and uptake the extracellular available cobamides for the microorganisms to maintain their dehalogenation ability. This pattern may be a common strategy that helps them sustain their normal metabolic activity and avoid the negative effects of cobamide deficiency for corrinoid-auxotrophic OHRB.

**Figure 4:**
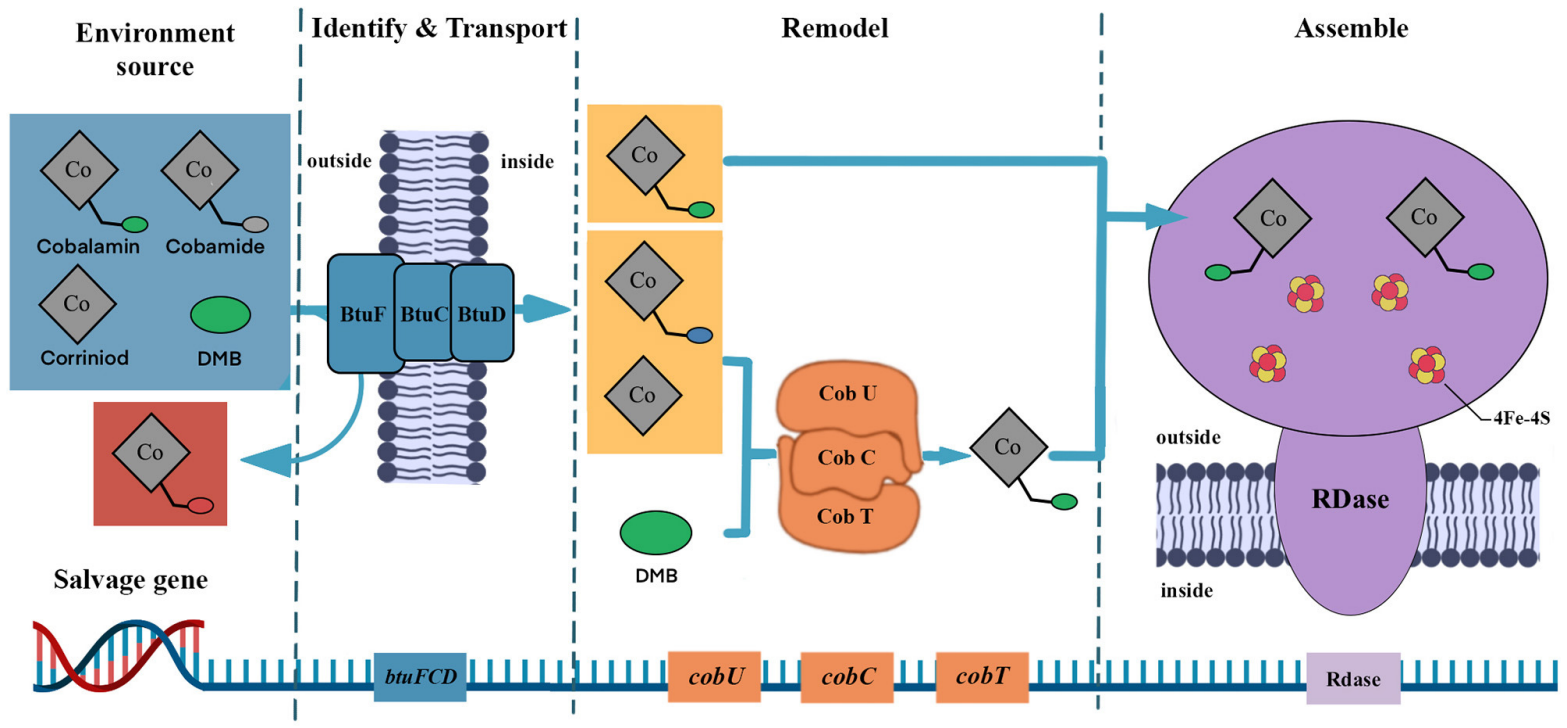
The salvage gene schematic. The blue section represents various cobamide types, the gray section represents the non-remodel cobamides type, and the red sphere is the immutable lower ligand, which cannot be remodeled. The yellow section represents remodeled cobamides, and the green sphere is the variable lower ligand, which can be directly used for RDase assembly after remodeling.

The biosynthetic cobamide has been found to show many structures. The function of cobamide-dependent enzymes depends on the core of cobamide upper ligands, lower ligands, corrin ring, and the nucleotide loop (Shelton et al., 2019). The corrinoid-auxotrophic OHRB can only use the special cobalamin for dehalogenation, while other cobamides cannot be used directly and need further remodeling. Compared with the direct use of cobalamin, the structure modification process is longer and limits the dechlorination rate, which leads to RDase activity at a minimal level (Keller et al., 2014; Men et al., 2014). As a result, these OHRB express the salvage genes that strengthen the use of available cobamides (Figure 4). This mode indicates that species *Dehalococcoides mccartyi* can assemble cobalamin if the precursors (e.g., corrinoid, DMB) are present. Men et al. (2015) detected the salvage of *Dehalococcoides mccartyi* from [*p*-Cre]Cobamide (*p*-Cresol as a lower ligand) and further confirmed that the *Dehalococcoides mccartyi* up-regulates the salvage genes in cobalamin deficiency environment, and use DMB to modify other cobamides forms (Men et al., 2017). However, cobamide remodeling is a complex metabolic pathway that leads obligate OHRB to use optimum cobamide to conserve energy for growth (Men et al., 2017; Balabanova et al., 2021). This suggests that direct uptake of cobalamin offers more advantages, such as energy saving, higher efficiency, and shorter durations, than modifying other cobamide structures through the remodeling function. We believe these cobamide salvage genes and related functions assemble a complete dehalogenation function of corrinoid-auxotrophic OHRB.

In conclusion, cobamide participates in the organohalide respiration process and is regarded as a key coenzyme for halogen removal. The obligate OHRB possess the functional genes that include cobamide uptake, transfer, and salvage, as well as remodeling functions that assist in the acquisition of cobamide from the environment and modify cobamide into a suitable structure; we summarize these examples in Table 2. This avoids synthesizing corrinoid *de novo*, which allows these obligate OHRBs to maintain dehalogenation and save energy efficiently. While facultative OHRB integrates many functions, efficient growth but low cell yield leads to lower dehalogenation efficiency and longer dehalogenation duration.

**Table 2:** Typical OHRB and their pathways for cobamide supplying.

**OHRB strain**	**Type**	**Organohalide**	**Optimal cobamide structure**	**Source of cobamide**	**Functional deficiency**	**Reference(s)**
*Dehalococcoides mccartyi* spp.^a^	Obligate	TCE; PCBs	Cobalamin	Salvage and remodel	No cobamide synthesis	(Yan et al., 2013, 2018; Chen et al., 2024; Zou et al., 2024)
*Dehalogenimonas* spp.^b^	Obligate	VC; DCF; 1,2-DBA; 1,2-DCA	Cobalamin	Salvage and remodel	No cobamide synthesis	(Yang et al., 2017; Li et al., 2022; Palau et al., 2023; Salom et al., 2023)
*Dehalobium* spp.	Obligate	PCBs; PCE	Cobalamin	Salvage and remodel	No cobamide synthesis	(Xu et al., 2023)
*Dehalobacter restrictus* spp.^c^	Obligate	PBDEs CF DCM	Cobalamin	Salvage and remodel	Incomplete cobamide synthesis	(Rupakula et al., 2013; Moore and Escalante-Semerena, 2016; Bulka et al., 2023; Kim and Han, 2024)
*Geobacter lovleyi* strain SZ	Facultative	PCE; TCE	Cobalamin	*De novo* synthesis	NA	(Nakamura et al., 2018; Zhong et al., 2024)
*Anaeromyxobacter dehalogenans*	Facultative	2-CP; 2,6-DCP; 2,5-DCP; 2-BrP	NR	Salvage	Cannot synthesis cobamide and remodel	(Sanford Robert et al., 2002; Moore and Escalante-Semerena, 2016)
*Desulfovibrio* sp.	Facultative	2-CP; PCE	Cobalamin	NR	NA	(Drzyzga and Gottschal Jan, 2002; Song et al., 2015)
*Sulfurospirillum multivorans*	Facultative	PCE; TCE	[Ade] cobamide	*De novo* synthesis	NA	(Kruse et al., 2021; Zhang et al., 2024)
*Desulfitobacterium spp*.^d^	Facultative	PCE; 2,3-DCP	Cobalamin; [5-OMeBza] cobamide	*De novo* synthesis	No cobamide transport	(Schubert et al., 2019; Lu et al., 2024)

^a^strain CBDB1, GT, BAV1, 195, and VS. ^b^strain GP, DCF, BL-DC-9. ^c^strain PER-K23, DSM 9455. ^d^strain Y51, DCB-2. ^e^Heterologous expression test. TCE, trichloroethylene; PCBs, polychlorinated biphenyls; VC, vinyl chloride; DCF, diclofenac; 1,2-DBA, 1,2-dibromoethane; 1,2-DCA, 1,2-dichloroethane; PBDEs, polybrsssominated diphenyl ethers; CF, chloroform; DCM, dichloromethane; 2-CP, 2-chlorophenol; 2,6-DCP, 2,6-dichlorophenol; 2,5-DCP, 2,5-dichlorophenol; 2-BrP, 2-bromophenol; 2,3-DCP, 2,3-dichlorophenol; NR, not reported; NA, not applicable.

## Microbial interactions for cobamides

Cobamides, H_2_, and carbon sources are the microbial interact substances in dehalogenating microbial communities. Cobamide is also an essential cofactor for several important enzymes catalyzing transmethylation and rearrangement reactions in bacteria and archaea. Cobamide can be key in facilitating cross-feeding and symbiosis (Feng et al., 2018; Sokolovskaya et al., 2020). Many bacteria and archaea can synthesize cobamide *de novo* and contain the cobamide salvage pathway (Fang et al., 2017; Agarwal et al., 2019; Kipkorir et al., 2021).

In the dehalogenation community, OHRB play the main role in reductive dehalogenation. The electron acceptor, electron donor, and electron transfer chain have been extensively studied, and cobamides are considered to be directly in charge of the removal of halogens (Wang et al., 2018, 2019; Cui et al., 2021). The corrinoid-auxotrophic OHRB relies on cobamide exchange with other species to sustain organohalide respiration, as a cobamide deficiency can disrupt the electron transport chain. This underscores the importance of a credible dehalogenation co-culture model, where microorganisms collaborate to provide essential substances, such as carbon sources, hydrogen, and cobamides. This forms a synergistic effect to enhance the dehalogenation performance and extent. Numerous studies have demonstrated that the OHRB and specific microbes, such as *Desulfovibrio* and *Methanosarcina* (Men et al., 2012; Wang et al., 2019), can establish an interspecies electron transfer mechanism that promotes the process of reductive dehalogenation, which is summarized in Table 3.

**Table 3:** Summary of co-cultures for reductive dehalogenation and interaction for cobamides.

**Co-culture**		**Interaction role**	**Supplement substance**	**Dehalogenation**	**Reference(s)**
**Corrinoid auxotrophs OHRB** ^a^	**Interaction member**				
*Dhc* BAV1	*Geobacter lovleyi*	Provide cobalamin	Without cobamide	PCE to ethene	(Yan et al., 2012)
*Dhc* FL2				PCE to VC	
*Dhc* BAV1	*Geobacter sulfurreducens*	Provide non-cobalamin	DMB	cis-DCE to ethene	
*Dhc* FL2				TCE to VC	
*Dhc* 195	NA	NA	Without cobamide	NS	(Men et al., 2014)
	NA	NA	≈ 100 μg L^−1^ Cobalamin	TCE to ethene	
	*Pelosinus fermentans* R7	Provide [Phe]cobamide	DMB	TCE to ethene (slow)	
*Dhc* BTF08	*S.multivorans*	Provide [Ade]cobamide	Without cobamide	NS	(Kruse et al., 2021)
			≈ 200 μg L^−1^ Cobalamin	PCE to ethene	
			DMB	PCE to ethene	
*Dhc* 195	*S.multivorans*	Provide [Ade]cobamide	Without cobamide	PCE to VC	(Kruse et al., 2021)
			≈ 200 μg L^−1^ Cobalamin		
			DMB		
*Dhc* BAV1	*M. barkeri* strain Fusaro	Provide [5-OHBza]cobamide	Without cobamide	cis-DCE to VC (slow)	(Yan et al., 2013)
			25 μg L^−1^ Cobalamin	cis-DCE to ethene	
			DMB	cis-DCE to ethene	
*Dhc* GT	*M. barkeri* strain Fusaro	Provide [5-OHBza]cobamide	Without cobamide	NS	(Yan et al., 2013)
			25 μg L^−1^ Cobalamin	NS	
			DMB	TCE to ethene	
*Dhc* FL2	*M. barkeri* strain Fusaro	Provide [5-OHBza]cobamide	Without cobamide	NS	(Yan et al., 2013)
			25 μg L^−1^ Cobalamin	NS	
			DMB	TCE to ethene	
*Dhc* BAV1	*Sporomusa ovata*	Provide [Phe]cobamide and [*p*-Cre]cobamide	Without cobamide	cis-DCE to VC (slow)	(Yan et al., 2013)
			25 μg L^−1^ Cobalamin	cis-DCE to VC	
*Dhc* BAV1	*Sporomusa* sp. strain KB-1	Guide biosynthesis cobalamin	Without cobamide	cis-DCE to VC (slow)	(Yan et al., 2013)
			DMB	cis-DCE to VC	
*Dhc* GT	*Sporomusa* sp. strain KB-1	Guide biosynthesis cobalamin	DMB	TCE to ethene	(Yan et al., 2013)
*Dhc* FL2	*Sporomusa* sp. strain KB-1	Guide biosynthesis cobalamin	DMB	TCE to ethene	(Yan et al., 2013)
*Dhc* 195	NA	NA	1 μg L^−1^ cobalamin	PCE to ethene	(He et al., 2007)
	*Desulfovibrio desulfuricans*	NR		PCE to VC and ethane (slow)	
	*Desulfovibrio desulfuricans* and *Acetobacterium woodii*	Provide cobalamin		PCE to VC and ethane	
SANAS culture (contains *Dhc* spp.)	family *Veillonellaceae*	Provide [*p*-Cre]cobamide	Without cobamide	TCE to VC and ethene	(Men et al., 2015)
	Other species	Provide cobamide			
YH Culture (contains *Dhc* spp.)	*Desulfovibrio, Clostridium, Geobacter*, and methanogens	Provide corrinoid	Without cobamide	TCE to DCE	(Wen et al., 2020)
			100 μg L^−1^ cobalamin	TCE to ethene	
			DMB	TCE to ethene	
*Dhbrestrictus* ^b^	*Sedimentibacter*	Provide cobamide	NR^b^	β-HCH to benzene and CB	(Maphosa et al., 2012)

^a^*Dhc, Dehalococcoides mccartyi*; *Dhb, Dehalobacter*. TCE, trichloroethylene; cis-DCE, cis-1,2-dichloroethylene; VC, vinyl chloride; DMB, 5,6-dimethyl benzimidazole; β-HCH, β-hexachlorocyclohexane; CB, chlorobenzene; NR, not reported; NA, not applicable; NS, not significant dehalogenation.

Facultative OHRB interacting with obligate OHRB is a common form. The dehalogenation community consists of OHRB with different metabolic patterns and niches. Some facultative OHRB can synthesize cobamide and dehalogenate organohalides, forming a more self-sufficient co-culture system than only obligate OHRB (Lai and Becker, 2013; Fincker and Spormann, 2017; Ning et al., 2022). While obligate OHRB has a stricter metabolism pathway, it depends on organohalides as electron acceptors, hydrogen or simple organic compounds (e.g., acetate) as electron donors, and other factors for their organohalide respiration. Although they have specific and efficient dehalogenation capabilities, the growth rate of the cell culture is slow, and the cultivation conditions are strict. As the genus *Dehalococcoides* lacks the gene to synthesize cobalamin, the growth of isolated *Dehalococcoides* is relatively slow, with a long doubling time (Men et al., 2013). In contrast, previous studies have demonstrated that *Dehalococcoides* in microbial enrichments or with sufficient cobalamin have a faster and more robust growth rate, reaching two times the cell density of cultures with limited cobalamin (Yan et al., 2013). When *Dehalococcoides* are co-cultured with other OHRB, their cell yield increases by 1.5 times compared to the control (Amos et al., 2009; Hug et al., 2012). These results suggest that *Dehalococcoides* can benefit from the interactions with other microorganisms and the supplementation of cobalamin from the environment.

As previously discussed, cobalamin is the most popular cofactor compared to other types of cobamides for most OHRB; therefore, microorganisms that have the ability to synthesize cobalamin are more likely to be effective partners in the dehalogenation process. It has been observed that both *G.lovleyi* and *G.sulfurreducens* can produce distinct cobamides. However, when these two were co-cultured independently with *Dehalococcoides*, only *G.lovleyi* supported reductive dehalogenation activity (Yan et al., 2012). The *G.lovleyi* directly released cobalamin as a suitable cofactor for reductive dehalogenation, whereas *G.sulfurreducen* released an unavailable cobamide, which could not support reductive dehalogenation activity. Providing cobalamin to *Dehalococcoides* is a highly effective way to promote reductive dehalogenation, and the co-culture strategy has been extended to combine the facultative OHRB to provide cobalamin. Some facultative OHRB, such as *Geobacter* sp. and *Dehalobacter* sp. can synthesize cobalamin and perform organohalide respiration. Wagner et al. (2012) suggested that an active *Geobacter lovleyi* community could provide *Dehalococoides* specific cobamide to establish a co-culture system. Consequently, obligate and facultative OHRB can potentially form a stronger dehalogenation community and eliminate the cobalamin restriction on obligate OHRB with simultaneous coupling of the dehalogenation process. From the perspective of cofactors, cobalamin is crucial for RDase due to its high reducibility, which facilitates the energy cycle of OHRB.

However, similar dehalogenation activity was observed in the OHRB community without exogenous cobalamin (Men et al., 2013). This confirms that other microorganisms provide cobalamin directly or other cobamide to remodel for *Dehalococcoides* during organohalide respiration. Despite the *Dehalococcoides* strain's incapacity to biosynthesis cobalamin, the cobalamin gap in OHRB supply and demand can be filled by other microorganisms.

Another microbial interaction involves non-OHRB cobamide producers, such as fermentors, acetogens, and methanogens. Known cobamide producers include *Clostridium* spp., *Desulfovibrio* spp., *Acetobacterium woodii*, and *Methanosarcina barkeri*, but their interspecific cobalamin transfer ability needs to be further confirmed (i.e., the ability to release cobalamin to the environment) (Hazra et al., 2015; Shelton et al., 2019). These anaerobic microorganisms can synthesize corrinoids *de novo* and export the cobamide to narrow the demand gap. Therefore, the corrinoid or cobamide supplier for corrinoid-auxotrophic OHRB within co-culture microbial communities must be explored further.

Furthermore, the co-culture system can enhance the dehalogenation performance of OHRB by modulating the interactions among dehalogenating communities (Min et al., 2019). Maphosa et al. (2012) reported that the *Sedimentibacter* strain provides corrinoid to *Dehalobacter* strain E1, addressing the deficiency in corrinoid synthesis. This suggests that these microorganisms play a key role in sustaining high rates of dehalogenation functions of OHRB. For example, methanogens can produce cobalamin during energy metabolism, which may be released from the cell and applied by corrinoid-auxotrophic OHRB for dechlorination (Wen et al., 2015). However, it should be noted that both methanogens and obligate OHRB utilize hydrogen in their metabolic processes and potentially can be competitors of each other. Meanwhile, interspecific competition is inevitable. Previous studies have reported that only about 5% of hydrogen was used as electron donors for organohalide respiration (Ma et al., 2003; Yang et al., 2020a), and large amounts of hydrogen were used for methanogenesis. In addition, it has been quantified that methanogenesis consumes about 80% of the hydrogen (Kuroda and Watanabe, 1995; Jiang et al., 2023). Although the hydrogen competitions actually happen between methanogens and OHRB, it has been accepted that the hydrogen demand between them is not at the same level (Feldewert et al., 2020). Previous studies have suggested that the dehalogenation of OHRB will be affected only when the hydrogen concentration in water is lower than 2 nM, but methanogens cannot consume hydrogen to this extent (Yang et al., 2020a). It is more likely that, compared to the disadvantage of hydrogen competition, methanogens have a greater positive impact by supporting reductive dehalogenation by providing cobalamin and other cofactors (Maymó-Gatell et al., 1995; Jin et al., 2020). Furthermore, other research shows that the methanogen F_430_ enzyme is similar to cobamide; the core Ni^+^ ion also contains high reducibility. Yuan et al. (2021) suggest that the MCR enzyme (Methyl-coenzyme M reductase) can reduce the activation barriers for dichlorination, which is a cobamide-similar structure. Therefore, methanogens are more beneficial for reductive dehalogenation than disadvantages.

Similarly, Li et al. (2021) reported a tri-culture system with *Shewanella oneidensis* MR-1, methanogens, and *Dehalococcoides mccartyi* strain 195 (*Dhc* 195) that established a high-efficiency electron transport network to assist TCE degradation (Li et al., 2019). MR-1 facilitates direct interspecies electron transfer (DIET) between community members, promoting methanogens and other members to synthesize cobalamin and accelerating the process of electron transfer to RDase. It is similar to the electron shuttles, which assist the interspecies electron transfer process. This indicates a feasible scheme for supporting dehalogenation, the free electron thought DIET combined with high-valence cobalamin (Co [II/III] state) to revert the high reducibility and finally supporting the *Dhc* 195 synthetic/activation RDase to enhance the organohalide respiration. In addition, the methanogens (e.g., *Methanosarcina barkeri, Methanobacterium formicicum, Methanobrevibacter ruminantium*, etc.) can synthesize 5′-hydroxybenzimidazolyl-cobamide (5-OHBza) and [Ade]cobamide, respectively, which could support the reductive dehalogenation of *Dehalococcoides* as well, they are potential cobalamin providers (Stupperich and Kräutler, 1988; Wagner et al., 2016). Studies have evaluated the association between methanogens and OHRB, and methanogens are not the only source of cobamide for OHRB (Yoshikawa et al., 2021; Yuan et al., 2021). However, there are reports that *Dehalococcoides* compete with methanogens for free cobamide (Wen et al., 2020), they may have other beneficial effects on the dehalogenation process, such as electron transfer, sustaining the low redox potential, or reducing energy barriers. Therefore, methanogens play a positive role in the dehalogenation community.

Additionally, a tri-culture of cobamide-producing bacteria with OHRB has also been reported; *Desulfovibrio vulgaris* Hildenborough (DVH) can produce cobalamin and establish a tri-culture system containing *Dhc* 195, DVH, and methanogens (Men et al., 2012). The *Dhc* 195 cell density in the tri-culture was approximately twice as high as in the isolated culture, and the expression of corrinoid transport and salvage function genes was decreased. This could be attributed to DVH, which provides cobalamin directly to *Dhc* 195, decreases structure remodeling energy consumption, and improves the effective corrinoid transfer, resulting in more energy conversion to support OHRB and a high cell yield. Moreover, Yan et al. (2013) used acetogens to establish a co-culture with *Dehalococcoides*: acetogens *Sporomusa ovata* and *Sporomusa* sp. KB-1, which can synthesize [Phe]cobamide and [*p*-Cre]cobamide, respectively. The [Phe]cobamide successfully activates organohalide respiration of *Dehalococcoides*, while [*p*-Cre]cobamide cannot. This indicates that the dehalogenation metabolism requires a specific cobamide type that the OHRB can selectively utilize before resorting to the remodeling function. However, this selection is strain-dependent and may not always occur. In that case, OHRB will start to transform cobamides and activate the remodeling function.

There are also microbial interaction modes that provide the lower ligand for remodeling. The remodeling function mechanism is designed to convert the lower ligand and produce sufficient cobalamin. It is plausible that the DMB is another key factor in the reductive dehalogenation of obligate OHRB. The remodeling function operates only when DMB is added to the co-culture microcosms as the lower ligand to guide cobalamin biosynthesis. However, DMB is mainly synthesized artificially, and there are few reported anaerobic biosynthetic pathways. Hazra et al. (2015) reported that *Eubacterium limosum* has a complete pathway of DMB biosynthesis. The anaerobic biosynthesis of DMB requires additional modification through the *bzaABCDE* genes. Shelton et al. (2019) suggest that this complete gene set is found only in a few species. It is still unclear which bacteria can biosynthesize or release DMB in an anaerobic dehalogenation community, but it was observed that DMB is the key lower ligand of cobalamin and can be applied to remodel cobamide into cobalamin. In addition, the DMB related utilization gene has been confirmed in the *Dehalococcoides* genome. *Dehalococcoides* can assemble cobalamin *in vitro* or *in vivo* by remodeling function, and the DMB synthesizer can be an efficient co-culture partner (Men et al., 2017; Esken et al., 2020; Mathur et al., 2020; Sokolovskaya et al., 2020).

In conclusion, we have summarized the interactions within dehalogenation communities, emphasizing the irreplaceable role of cobamide in symbiosis. We have noted the capacity of numerous bacteria and archaea to synthesize cobamides and the demand within the co-culture. Furthermore, we have discussed the positive impact of symbiotic partners in promoting reductive dehalogenation, such as methanogens, facultative OHRB, DVH, and acetogens. The significance of DMB in the remodeling function as the key ligand of cobalamin is emphasized. Despite the limited knowledge regarding DMB biosynthesis, it is confirmed that the incorporation of DMB enhances dehalogenation capacity, and further study is required.

## Conclusion and perspective

Obligate and facultative OHRB play a vital role in microbial ecosystems, occupying an irreplaceable role in dehalogenation. The reductive dehalogenation process has been accepted as the optimal pathway for reducing halogenated compounds, with cobamide identified as the core component. Except the *Sulfurospirillum multivorans* directly use the [Ade]cobamide, cobalamin has been proposed as the optimal structure for reductive dehalogenation. Cobalamin-mediated mechanisms have a similar electron flow in reductive dehalogenation. Furthermore, obligate and facultative OHRB have different adaptations in corrinoid-auxotrophic environments. These corrinoid-auxotrophic OHRBs, through salvage and remodel pathway to narrow the gap of cobalamin deficiency, and establish a co-culture system with other cobamide producers for continuous reductive dehalogenation.

Current research is focused on determining the biosynthetic pathways of cobalamin in most OHRB, gene regulatory networks, and the effects of cobalamin on dehalogenation capacity under specific environmental conditions. However, the distinct degrees and rates of dehalogenation in the obligate and facultative OHRB within microbial ecosystems have been observed. Not only cobalamin, such as organohalide type, oxygen, temperature, and pH, competition, and interaction with other microorganisms also significant influence the rate and degree of dehalogenation. Therefore, it is important to further explore the factors that influence its species and abundance in specific environments. These factors can also guide the design and optimization of microbial communities to avoid the disadvantageous conditions encountered in the field. However, to forecast such complex microbial communities, which require significant amounts of data and suitable deep learning models, metatranscriptomics, and metaproteomics are necessary; more specifically, experimental information on microbial interaction should be provided. Furthermore, previous studies of the cobamide selectivity in OHRB are still in the laboratory stage. Field tests of community interactions for cobamides require further verification, and understanding the specific cobamide preferences of these microorganisms is crucial. However, the challenges of the lengthy culture time and scarcity of purified OHRB are significant. UsingAI-enabled environmental computing to narrow the scope of experiments is a viable option, which requires several of environmrntal data sets (e.g., substrate property, environmental and species information, interaction network, etc) for structured learning and deep learing to predict interspecific substrate exchange. The effect of different cobamides and their lower ligands on reductive dehalogenation may be elucidated through machine learning. The recently reported AlphaFold 3 accurately predicts macromolecules, and this may be used to predict the complex cobamides family and the ligand role in RDase which would promote the study of the binding and catalytic mechanisms of cobalamin.

In conclusion, future research in the field of OHRB should delve into their characteristics and mechanisms, focusing on cobalamin-related processes, interactions between microorganisms, symbiotic relationships, and their interactions with environmental factors. These can be facilitating by artificial intelligence and deep learning. The results of these studies will contribute to improving the application efficiency of OHRB in environmental remediation, thus facilitating the development of environmental remediation technologies.

## Author contributions

YLu: Conceptualization, Methodology, Visualization, Writing – original draft, Writing – review & editing. FL: Visualization, Writing – review & editing. JZ: Supervision, Writing – review & editing. QT: Writing – review & editing. DY: Conceptualization, Funding acquisition, Supervision, Writing – review & editing. YLi: Conceptualization, Funding acquisition, Project administration, Supervision, Writing – review & editing.
